# Rapid Detection of Resistance Mutations in Multidrug‐Resistant Tuberculosis With GenoType MTBDR*sl* Assay

**DOI:** 10.1155/pm/6993694

**Published:** 2026-07-07

**Authors:** Shaina Gaikwad, Antisha Tiwari, Jitendra Singh, Alkesh Kumar Khurana, Sagar Khadanga, Shashank Purwar, Debasis Biswas, Anand Kumar Maurya

**Affiliations:** ^1^ Department of Microbiology, All India Institute of Medical Sciences, Bhopal, Madhya Pradesh, India, aiims.edu; ^2^ Department of Translational Medicine, All India Institute of Medical Sciences, Bhopal, Madhya Pradesh, India, aiims.edu; ^3^ Department of Pulmonary Medicine, All India Institute of Medical Sciences, Bhopal, Madhya Pradesh, India, aiims.edu; ^4^ Department of General Medicine, All India Institute of Medical Sciences, Bhopal, Madhya Pradesh, India, aiims.edu

**Keywords:** drug resistance, GenoType MTBDR*sl*, multidrug-resistant tuberculosis (MDR-TB), *Mycobacterium tuberculosis* (Mtb)

## Abstract

**Background:**

Multidrug‐resistant tuberculosis (MDR‐TB) is becoming a major threat to the global control of tuberculosis (TB). The situation worsens when fluoroquinolone (FQ) and second‐line injectable (SLID) resistant strains are involved. This study assesses the utility of MTBDR*sl* V2.0 line probe assay (LPA) in identifying resistance patterns to FQ and SLID in MDR‐TB isolates.

**Methods:**

A total of 74 MDR‐TB isolates were tested for genetic mutations that confer resistance to FQs and against SLIDs by applying the GenoType MTBDR*sl* V2.0 LPA. This assay targets resistance‐associated mutations in the *gyrA* and *gyrB* genes for FQ resistance, and in the *rrs* and *eis* genes for resistance to aminoglycosides and capreomycin.

**Results:**

Of the 74 isolates examined, 14 (18.9%) had FQ resistance. Most of these isolates had mutations in the *gyrA* gene, most commonly at codon A90V (6.7%), followed by D94G (5.4%), and various D94 variations. No mutations were found in the rrs, gyrB, or eis genes, and no resistance to SLIDs was found. With no discernible contribution from *rrs, gyrB*, or *eis* genes, our results emphasize *gyrA* mutations, particularly A90V and D94G, as important molecular markers of FQ resistance.

**Conclusion:**

The MTBDR*sl* V2.0 assay demonstrated operational utility for rapid detection of resistance‐associated mutations related to FQs and SLID drugs in MDR‐TB isolates. Its rapid turnaround time expanded mutation coverage, and applicability in routine diagnostic settings make it a useful tool for molecular resistance profiling in high TB‐burden settings.

## 1. Introduction

Tuberculosis (TB), caused by *Mycobacterium tuberculosis* (Mtb), remains a significant global health concern, impacting mortality and morbidity rates [1]. Rapid detection of resistance‐associated mutations is important for optimizing treatment strategies and reducing transmission of drug‐resistant TB. Conventional phenotypic drug susceptibility testing is technically demanding and may require several weeks, whereas molecular methods offer a faster approach for identifying resistance‐associated mutations. Molecular methods targeting resistance‐related mutations enable timely diagnosis as they provide insight into the genetic mechanisms of resistance to drugs in Mtb [2]. Conventional phenotypic drug susceptibility testing is technically demanding and may require several weeks, whereas molecular methods provide more rapid detection of resistance‐associated mutations. Therefore, there is much focus on rapid molecular diagnostic techniques for identifying drug resistance.

Second‐line drug resistance can be detected with the help of the GenoType MTBDR*sl* assay kit, with Hain Lifesciences, Germany. Specifically, it uses multiplex PCR coupled with reverse hybridization, which can detect resistance in the *gyrA*, *rrs*, and *embB* genes for fluoroquinolone (FQ), CAP/AK/KAN/VIO, and ethambutol (EMB) [3]. The assay can be performed directly in clinical specimens or cultured isolates. MTBDR*sl* demonstrated high levels of accuracy in detecting resistance to FQs and second‐line injectable drugs, and showed satisfactory performance for EMB resistance [4]. The GenoType MTBDR*sl* Version 2.0 assay represents a significant improvement over earlier versions by expanding mutation detection, particularly with the inclusion of *gyrB* mutations for FQ resistance and *eis* promoter mutations associated with low‐level aminoglycoside resistance. These modifications improve diagnostic sensitivity and enable the identification of resistance mechanisms that were not sufficiently captured in earlier assay version. The purpose of this study was to evaluate the practical usability of the GenoType MTBDR*sl* V2.0 assay in standard diagnostic settings and to characterize the frequency and distribution of resistance‐associated mutations found in multidrug‐resistant tuberculosis (MDR‐TB) isolates.

## 2. Materials and Methods

### 2.1. Ethics Statement

The Institutional Ethics Committee (IEC) of AIIMS, Bhopal (IHEC‐LOP/2023/IL110) approved this study. This study was conducted in accordance with the principles of the Declaration of Helsinki and the principles of Good Clinical Practice.

### 2.2. Study Setting

Samples for line probe assay under the NTEP program were received in the laboratory between June 1, 2023, and December 31, 2023. The samples included both pulmonary tuberculosis (PTB) and extrapulmonary tuberculosis (EPTB) cases that tested positive by nucleic acid amplification tests (NAAT).

### 2.3. Type of Sampling and Reasons for Selection

The study used sampling in targeting the MDR‐TB isolates proven to ensure cases presenting resistance to first‐line drugs (rifampicin [RIF] and isoniazid [INH]). These isolates were selected strategically to support the study′s primary objective: to investigate resistance‐associated mutations that play a pivotal role in determining second‐line treatment regimens.

### 2.4. Inclusion Criteria

Patients with laboratory‐confirmed MDR‐TB, defined as resistance to both RIF and INH, were included in the study. Both PTB and EPTB cases were eligible. Newly diagnosed and previously treated MDR‐TB cases identified through routine diagnostic testing under the NTEP program were included.

### 2.5. Exclusion Criteria

Cases of drug‐sensitive TB and mono‐resistant TB were excluded because the study specifically aimed to evaluate resistance‐associated mutations in confirmed MDR‐TB isolates. Isolates lacking confirmed resistance to both RIF and INH were excluded to maintain a homogeneous study population relevant to the study objectives. Patients with prior exposure to second‐line anti‐TB drugs were excluded to reduce potential confounding related to acquired resistance patterns. Samples collected outside the defined study period or geographical setting and cases lacking appropriate ethical approval were also excluded. Although MTBDR*sl* V2.0 can be performed on both smear‐positive and smear‐negative specimens according to manufacturer recommendations, only smear‐positive and/or NAAT‐positive samples were included to ensure adequate bacillary load and minimize indeterminate results.

### 2.6. Laboratory Work

#### 2.6.1. Sample Decontamination Processing

All collected samples were sent to the mycobacteriology laboratory for line probe assay testing as per NTEP guidelines. The 4% N‐acetyl‐L‐cysteine/sodium hydroxide (NALC/NaOH) method was used to decontaminate these samples. Such an approach facilitates the removal of unwanted materials such as normal flora and other media contents before the actual downstream analysis. The NALC‐NaOH‐sodium citrate approach was used to carry out the decontamination process. The mixture was prepared by combining 0.5 g of NALC with an equal quantity of 2.9% sodium citrate and 4% NaOH in 100 mL of a sodium citrate‐NaOH solution. The mixture was then added to a sterile, 50 mL plastic centrifuge tube containing the sample. After vortexing for about 20 s, the tube was left to stand for 20 min. The mixture was then swirled manually after adding sterile phosphate buffer solution (pH 6.8) to each tube up to the 50 mL mark, the mixture was manually swirled. Following a 15‐min 3000‐g speed centrifugation of the samples, the supernatant was disposed of. Finally, the pellet was suspended in a phosphate buffer solution with a pH of 6.8 to reach a volume of 1 mL, after which it was sent for further processing.

#### 2.6.2. Acid‐Fast Bacilli (AFB) Staining

Once decontamination of the samples was completed, AFB smear slides were prepared for microscopic evaluation. To stain the specimens, the Ziehl–Neelsen method was employed. Scanty, 1+, 2+, and 3+ were the evaluations given to the smears for 1–10 AFB/100 oil emulsion fields (OIF), 10–99 AFB/100 OIF, and > 10 AFB/OIF as per NTEP guidelines. For each case, three repeats were carried out.

#### 2.6.3. GenoType MTBDR*sl* Assay

The MTBDR*sl* assay is based on DNA Strip technology [5] and is performed in three key steps: (i) DNA extraction from decontaminated sputum samples or cultured materials; (ii) multiplex amplification carried out using biotinylated primers; and (iii) reverse hybridization aimed at detecting target sequences.

i. DNA Extraction. The decontaminated samples were then subjected to DNA extraction using a GenoLyse kit (Hain Lifescience, Nehren, Germany). In brief, 1000 *μ*L of the decontaminated sample was centrifuged for 15 min at 10,000 × g. The pellet was again resuspended in 100 *μ*L of lysis buffer and vortexed for 30 s. After that, tubes were kept in a sonicator for 5 min, and then this suspension was incubated at 95°C in a water bath. Next, the neutralizing buffer was added to the mixture (100 *μ*L). The mixture was then vortexed for 30 s and subsequently subjected to a centrifugation process at 13,000 × g for 10 min. DNA was collected from the supernatant and processed for PCR amplification.
ii. PCR and reverse hybridization. The extracted DNA was directly from the decontaminated samples subjected to the detection of MTBC and resistance to FQs and second‐line injectable drugs (SLIDs), according to the manufacturer′s instructions (Hain Lifescience, Nehren, Germany). Briefly, after PCR, the PCR amplicons were hybridized to certain oligonucleotide probes that were fixed on the strip. Six control bands are present on the strip to ensure that the test protocol is verified. These bands include a conjugate control (CC) band, an amplification control (AC) band, a band tailored for the Mtb complex, and three locus control bands for drugs *gyrA* for FQs, *rrs* for amikacin (AMK), and *embB* for EMB. If each control zone is present on the strip, the outcome is considered valid. When all wild‐type (WT) probes for the corresponding gene stain positively and no mutant (MUT) probe hybridizes, the isolate is considered “sensitive” to that drug. In contrast, if one or more WT probes are missing and/or a MUT probe is present, the isolate is considered “resistant” [6].

### 2.7. Statistical Analysis

Descriptive statistics were used in the analysis of the data. The frequencies and percentages of mutations associated with resistance to FQs and second‐line injectables were calculated. Results were summarized to provide an overview of the resistance patterns observed.

## 3. Results

A total of 74 MDR‐TB patients were included in the SL‐LPA study, as presented in Table 1. Males constituted a higher proportion (62.1%) than females (37.8%), and the predominant age group was 20–40 years (47.2%). Most patients (85.1%) presented with PTB, and the majority were treatment‐naive cases (79.7%). Pulmonary specimens demonstrated comparatively higher detection rates than extrapulmonary specimens, whereas MTBDR*sl* V2.0 was successfully performed on both sample types.

**Table 1:** Comprehensive profile of MDR‐TB patients: demographics, clinical characteristics, and outcomes.

Variable	Category	Frequency (*n* − 74)	Percentage (100%)
Gender	Male	46	62.1%
	Female	28	37.8%
Age (Years)	< 20	11	14.8%
	20–40	35	47.2%
	41–60	23	31.08%
	61–80	5	6.7%
Sample type	Pulmonary samples	63	85.1%
	Extra pulmonary samples	11	14.8%
Treatment	Treatment‐naive	59	79.7%
	Previously treated	15	20.2%
HIV status	Negative	67	90.5%
	Unknown	7	9.4%
Smoking status	Smoker	11	14.8%
	Nonsmoker	60	81.08%
	Unknown	3	4.05%
Alcohol consumption	Yes	2	2.7%
	No	69	93.2%
	Unknown	3	4.05%
Diabetic status	Yes	4	5.4%
	No	67	90.5%
	Unknown	3	4.05%
Tobacco consumption	Yes	26	35.1%
	No	45	60.8%
	Unknown	3	4.05%
TB history in family	Yes	5	6.7%
	No	66	89.1%
Microbiological criteria	Unknown	3	4.05%
	1+	7	9.4%
	2+	5	6.7%
	3+	9	12.1%
	Positive	18	24.3%
	Scanty	2	2.7%
	Negative	33	44.5%
Confirmed cases	MDR‐TB	74	100%
Treatment outcome	Treatment completed	46	62.1%
	Treatment ongoing	23	31.08%
	Death	5	6.7%

HIV negativity was noted in 90.5% of the patients. Lifestyle factors such as smoking accounted for 14.8% of cases, whereas alcohol consumption and tobacco use were 2.7% and 31.5%, respectively; however, the reporting rate was quite high for unknown cases. Microbiological testing indicated that 24.3% of cases showed positive smear grading, and the demographic and clinical characteristics of the study population are summarized in Table 1. Treatment outcomes showed 62.1% completion, with ongoing treatment and mortality rate of 31.08% and 6.7%, respectively. The demographic and microbiological characteristics of the study population are summarized in Table 1.

**Figure 1:**
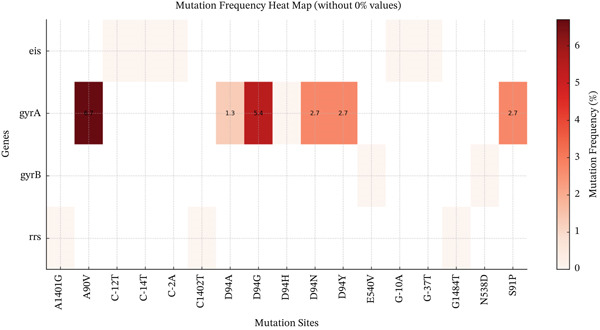
Mutation frequency heat map of drug‐resistance–associated loci in Mtb isolates. The heat map depicts the percentage frequency of observed mutations (excluding 0% values) across key genes—*eis*, *gyrA*, *gyrB*, and *rrs*—and their respective mutation sites. The color intensity corresponds to the mutation frequency (%) as indicated by the adjacent scale bar, with darker shades representing higher frequencies.

A mutation frequency heat map (Figure 1) was generated to visualize the distribution of resistance‐associated mutations across the *eis*, *gyrA*, *gyrB*, and *rrs* genes. Only mutations detected at nonzero frequencies were plotted. Among the analyzed loci, *gyrA* showed the highest frequency of mutations, predominantly A90V (6.7%) and D94G (5.4%), followed by D94A (1.3%), D94N, D94Y, and S91P (2.7% each). No mutations were detected in *eis*, *gyrB*, or *rrs*. The gradient color scale indicates mutation frequency, with darker shades representing higher frequencies. Because this study did not perform a parallel phenotypic drug‐susceptibility test (e.g., MGIT/LJ proportion method) or sequencing as a reference standard, we did not calculate or report diagnostic accuracy measures (sensitivity, specificity, PPV or NPV) for the assay within this cohort—the figures/tables, therefore, present SL‐LPA detection frequencies (counts and percentages) only.

**Figure 2:**
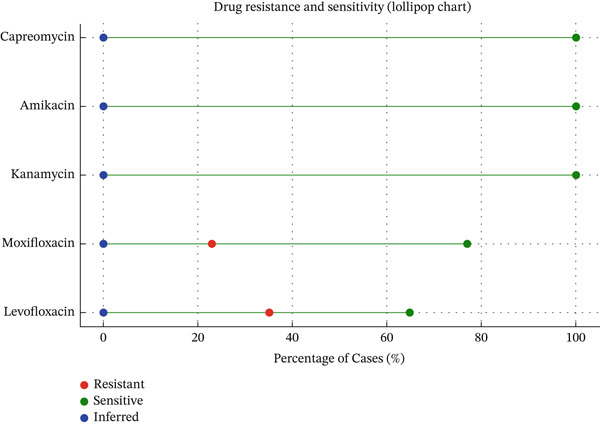
Lollipop chart summarizing the number of cases that were resistant (red), no resistance‐associated mutations detected (green), or inferred (blue) to each second‐line drug (capreomycin, amikacin, kanamycin, moxifloxacin, and levofloxacin).

A lollipop chart (Figure 2) displays the distribution of drug susceptible and resistance among the tested isolates. No resistance‐associated mutations related to kanamycin, amikacin, or capreomycin were detected. Although the remaining isolates for the FQs were primarily showed no resistance‐associated mutations (77.02% for moxifloxacin and 64.8% for levofloxacin), moxifloxacin and levofloxacin resistance was found in about 22.9% and 35.1% of cases, respectively. The blue markers indicate absence of resistance‐associated mutations for each drug. Resistance to FQs was observed more frequently than resistance to aminoglycosides and capreomycin in the analyzed isolates.

## 4. Discussion

The present study indicates the importance of the MTBDR*sl* V2.0 assay in establishing resistance by detecting drug‐resistant mutations in Mtb isolates, most especially when the cases are those of MDR‐TB and pre‐XDR‐TB. Our results, obtained from a collection of 74 samples, underscore the need to use advanced molecular diagnostic tools so that resistance mutations that cannot be identified through conventional methods, such as smear microscopy and culture‐based techniques, can be detected.

The MTBDR*sl* V2.0 assay provides a rapid and effective method to detect mutations that confer resistance to second‐line drugs, such as FQs and injectables, which form important parts of the regimen for MDR‐TB and pre‐XDR‐TB [7]. It enables the identification of specific genetic mutations, which is useful for selecting appropriate treatment strategies in the management of DR‐TB. From a programmatic perspective, MTBDR*sl* v2.0 provides a number of useful benefits that facilitate its incorporation into standard TB diagnostic workflows. Highly specialized infrastructure is not necessary because the assay can be used in molecular laboratories that are already established under national TB programs like NTEP and are reasonably equipped. Strip‐based hybridization with uniform interpretation criteria is used in the relatively simple test method, which enhances usability and lowers operator‐dependent variability. The rapid turnaround time may enable faster treatment initiation and possibly reduce patient care delays compared with traditional phenotypic drug susceptibility testing. The assay may increase overall programming efficiency through quick resistance detection and treatment decision optimization, even though setup and operating costs may vary based on laboratory infrastructure, reagent procurement, and testing volume. However, the current study did not conduct a thorough cost analysis that included setup, operating, and cost‐per‐test assessment costs.

The MTBDR*sl* V2.0 assay offers several improvements over traditional phenotypic drug susceptibility testing and previous molecular tests, in addition to its ability to identify resistance‐associated mutations. Compared to culture‐based techniques, which can take several weeks, the assay yields data in 24–48 h, greatly lowering turnaround time. It requires a moderately equipped molecular laboratory and is easier to implement within existing TB diagnostic infrastructure under national programs such as NTEP. Strip‐based hybridization reduces operator dependence and technological complexity by enabling simple interpretation. Additionally, early diagnosis and prompt initiation of appropriate medication improve overall cost‐effectiveness by reducing transmission and treatment delays, even though initial setup costs may be higher. Significantly, compared to previous iterations, MTBDR*sl* v2.0′s enhanced mutation coverage improves its diagnostic yield, especially in identifying resistance mechanisms that could otherwise go undiscovered. In recent years, newer molecular diagnostic platforms such as XDR‐Xpert and targeted next‐generation sequencing (t‐NGS) have further advanced the detection of drug‐resistant TB [8]. In the case of t‐NGS, these technologies provide comprehensive mutation profiling across multiple resistance‐associated genes, along with quick turnaround times. However, because they are more expensive, need sophisticated infrastructure, and demand technical know‐how, their general use in ordinary programmed contexts is still restricted. The MTBDR*sl* V2.0 test, on the other hand, offers a balanced strategy by providing quick and accurate identification of key resistance mutations at a relatively lower operational complexity and expense. A comparison of MTBDR*sl* V2.0 with other molecular diagnostic platforms used for the detection of drug‐resistant TB is presented in Table **2**. Although XDR‐Xpert and t‐NGS are valuable in specialized or reference laboratory settings, MTBDR*sl* V2.0 remains a more feasible and scalable option for integration into national TB control programs, particularly in resource‐limited, high‐burden regions.

**Table 2:** Comparison of MTBDR*sl* V2.0 with other molecular platforms used for detection of drug‐resistant tuberculosis [8–11].

Feature	MTBDR*sl* V2.0	XDR‐Xpert	CBNAAT/TrueNat	Targeted NGS (t‐NGS)
Primary purpose	Detection of second‐line drug resistance mutations	Rapid detection of XDR‐associated resistance	Initial TB detection and rifampicin resistance screening	Comprehensive mutation profiling
Target genes	*gyrA*, *gyrB*, *rrs*, and *eis*	Multiple resistance‐associated genes	Mainly *rpoB* (RIF resistance)	Multiple resistance‐associated genes simultaneously
Drug resistance coverage	FQ and SLID resistance	Expanded resistance profile	Primarily rifampicin resistance	Broad resistance profile across multiple drugs
Turnaround time	~24–48 h	~1–2 h	~1–2 h	Several days
Infrastructure requirement	Moderate molecular laboratory setup	Cartridge‐based platform	Minimal–moderate setup	Advanced sequencing facility
Technical expertise	Moderate	Low	Low	High
Cost considerations	Moderate	Moderate–high	Lower–moderate	High
Programmatic feasibility	Suitable for routine TB programs	Suitable where infrastructure exists	Widely implemented	Mainly reference laboratories
Major advantage	Expanded mutation coverage with operational feasibility	Very rapid detection	Rapid screening and decentralization	Comprehensive mutation analysis
Limitation	Detects predefined mutations only	Limited mutation panel	Limited resistance profiling	Higher cost and technical complexity

Abbreviations: CBNAAT, cartridge‐based nucleic acid amplification test; FQ, fluoroquinolone; NGS, next‐generation sequencing; SLID, second‐line injectable drugs.

In the present study, all included smear‐positive and/or NAAT‐positive samples yielded valid MTBDR*sl* V2.0 results, including extrapulmonary specimens. Although a direct comparison with earlier assay versions was not performed, this observation suggests improved applicability of MTBDR*sl* V2.0 for direct testing on clinical specimens, particularly extrapulmonary samples, which traditionally posed challenges for earlier line probe assay versions and often required prior culture amplification. These findings further support the operational utility of MTBDR*sl* V2.0 in routine diagnostic settings.

The genotypic resistance patterns observed through the MTBDR*sl* assay in our study allowed for both the detection of resistance to key drugs and the identification of mutations associated with a poor prognosis. FQ resistance is primarily caused by mutations that occur in the quinolone resistance‐determining region (QRDR) of *gyrA*, whereas mutations within the *rrs* gene correlate with resistance to aminoglycosides and other injectable drugs.

We analyzed 74 MDR‐TB isolates and observed that 61 isolates showed no detectable resistance‐associated mutations. Mutations were mostly observed in the *gyrA* gene (18.9%). This rate of FQ resistance is consistent with cross‐sectional studies conducted in other high TB‐burden countries, where FQ resistance is currently seen as a significant problem in the treatment of MDR‐TB. The 100% absence of resistance‐associated mutations to SLIDs indicates that, despite the presence of FQ resistance, SLID resistance remains absent in this population. These mutational changes were found on the *rrs* and *eis* genes, which agree with other molecular epidemiology studies that have documented similar resistance profiles. Another study conducted by Dalal et al. from 2005 to 2013 studied eight healthcare facilities in the greater Mumbai area, investigated the trends in drug resistance over time in a cohort of individuals with MDR‐TB. Between 2005–2007 and 2011–2013, the percentage of patients with ofloxacin and moxifloxacin resistance rose significantly, rising from 60.0% to 69.5% and from 57.6% to 75.3%, respectively (*p* < 0.05) [12]. According to a meta‐analysis by Ho et al. [13], FQ resistance in TB is mostly limited to MDR strains worldwide. This resistance pattern′s global prevalence is currently unknown because most areas with endemic TB lack surveillance data.

Here, the magnitude of FQ among the samples was compared in this study; a fairly high 18.9% of the samples exhibited resistance to FQs, and no resistance to SLIDs was observed, indicating a 100% absence of resistance‐associated mutations rate in the study population. This comparison highlights that, unlike FQs, no resistance to SLIDs was observed in the isolates studied. In another study, Singh and Jain examined whether a shorter regimen was suitable for MDR patients in a programmatic setting, as part of a related trial conducted in northern India. Of the 541 conclusive results, nearly 50% of the strains for line probe assay‐second line drugs (LPA‐SLD) were resistant to FQs alone, whereas 8.3% were resistant to both FQs and SLIDs [14]. In the referenced study, the rate of mutations in *gyrA* related mutations associated with FQ resistance was observed at 78.3% [15], which is comparable to findings studies conducted in other settings [16]. The results agree with our study in assessing FQ resistance with the MTBDR*sl* V2.0 assay, where detection of such mutations has played a key role in elucidating mechanisms of resistance. From the findings in both studies, FQ resistance appears to be an increasing challenge in the MDR‐TB treatment, although SLIDs seem to remain relevant in the treatment of many patients with a far lower resistance level. Ajbani et al. [16] concluded in their study that 88.23% (150/170) of the participants reported a valid test overall: 100% for FQ (*gyrA*; 170/170), 94.11% for EMB (*embB*; 160/170), and 88.23% for second line injectables (*rrs*), whereas the undetermined test rate was 11.76% (20/170), falling within an acceptable range.

Among all identified genetic changes, the alterations of the *gyrA* gene are reported to be the most common cause of resistance to FQs, which are currently considered to be one of the most effective drugs against MDR‐TB. In patients with TB who possess such mutations, high‐level resistance to FQs is reported to have worsened treatment outcomes. In a study, analysis of treatment cohorts enrolled in Peru and with high levels of DR showed that *gyrA* mutations associate with poor outcomes, like death or failure of the treatment. When potential confounders such as patients′ severity of illness, other treatment factors, and newer‐generation FQs are considered, patients with these mutations continued to have a poorer prognosis [17].Accordingly, this study infers that the existence of *gyrA* mutations, especially those giving a high level of resistance, reduces the effectiveness of the treatment regimens. In addition, they received late‐generation FQs, which are generally more effective against divergent resistant strains, yet the treatment outcomes were poor. This supports the current poor treatment outcomes with FQ‐based regimens in settings with *gyrA* mutations. Therefore, the study emphasizes the importance of improving and adapting the management of such patients, possibly by using new or alternative drugs to counteract the resistance conferred by these genetic changes. The persistent problem of *gyrA* resistance serves as a reminder that molecular methods are necessary for early identification of these mutations, enabling more effective treatment adjustments in such cases.

The study lacks validation of the GenoType MTBDR*sl* assay′s ability to detect rare or novel resistance mutations; additionally, it suffers from a small sample size and geographic focus that might not aid in generalization. There are various limitations to the current investigation. First, resistance‐associated mutations found by MTBDR*sl* V2.0 were not cross‐validated by comparison with previous line probe assay versions or by employing phenotypic drug susceptibility testing techniques as MGIT‐DST. Furthermore, no sequencing‐based validation of the mutations found was carried out. Therefore, rather than being a clinical validation or diagnostic accuracy study, the study should be seen as a descriptive investigation of mutation frequencies. Additionally, the results may not be as broadly applicable due to the single‐center study design, relatively limited sample size, and exclusion of smear‐negative samples. However, the study offers valuable information about how resistance‐associated mutations are distributed and how MTBDR*sl* V2.0 can be used in standard diagnostic settings. Furthermore, treatment response analysis and comprehensive longitudinal clinical follow‐up were not carried out in this investigation. The study was not intended to correlate resistance‐associated mutations with therapeutic response or clinical outcomes, despite the fact that treatment category and outcomes were descriptively presented.

## 5. Conclusion

The current study concludes that the GenoType MTBDR*sl* V2.0 assay is useful for rapidly identifying resistance‐associated mutations linked to FQs and second‐line injectable drugs in MDR‐TB isolates. Shorter turnaround times, ease of use, increased mutation coverage, and suitability for both pulmonary and extrapulmonary specimens are among the assay′s operational benefits. A high frequency of *gyrA* mutations associated with FQ resistance was observed, whereas no resistance‐associated mutations were detected in the *rrs*, *gyrB*, or *eis* genes in the analyzed isolates. These results support the operational usability of MTBDR*sl* V2.0 in routine diagnostic settings, especially in resource‐constrained and high‐TB‐burden locations, and offer descriptive insights into mutation distribution patterns. To determine diagnostic accuracy and clinical relevance, however, more research utilizing sequencing‐based validation and phenotypic drug susceptibility testing is needed.

## Author Contributions

Conceptualization, A.K.M.; methodology, A.K.M. and S.G.; software, S.G. and A.T.; validation, S.G. and A.T.; formal analysis, S.G. and A.T.; investigation, A.K.M., J.S., A.K.K., and S.K.; resources, S.G. and A.T.; data curation, S.G. and A.T.; writing—original draft preparation, A.K.M. and S.G.; writing—review and editing, S.G., A.K.M., A.T., S.K., A.K.K., J.S., S.P. and D.B.; visualization, A.K.M. and J.S.; supervision, A.K.M., S.P., and D.B.; project administration, A.K.M.; and funding acquisition, A.K.M.

## Funding

No funding was received for this manuscript.

## Disclosure

The authors have reviewed and edited all AI‐assisted content and take full responsibility for the final publication.

## Conflicts of Interest

The authors declare no conflicts of interest.

## Data Availability

The data that support the findings of this study are available from the corresponding author upon reasonable request.
